# Using Lempel–Ziv Complexity to Assess ECG Signal Quality

**DOI:** 10.1007/s40846-016-0165-5

**Published:** 2016-10-05

**Authors:** Yatao Zhang, Shoushui Wei, Costanzo Di Maria, Chengyu Liu

**Affiliations:** 1 School of Control Science and Engineering, Shandong University, Jinan, 250061 People’s Republic of China; 2 School of Mechanical, Electrical & Information Engineering, Shandong University, Weihai, 264209 People’s Republic of China; 3 Institute of Cellular Medicine, Newcastle University, Newcastle upon Tyne, NE1 4LP UK; 4 Regional Medical Physics Department, Freeman Hospital, Newcastle upon Tyne, NE7 7DN UK

**Keywords:** Electrocardiography (ECG), Lempel–Ziv (LZ) complexity, Signal quality assessment, Signal-to-noise ratio (SNR)

## Abstract

The poor quality of wireless electrocardiography (ECG) recordings can lead to misdiagnosis and waste of medical resources. This study presents an interpretation of Lempel–Ziv (LZ) complexity in terms of ECG quality assessment, and verifies its performance on real ECG signals. Firstly, LZ complexities for typical signals, namely high-frequency (HF) noise, low-frequency (LF) noise, power-line (PL) noise, impulse (IM) noise, clean artificial ECG signals, and ECG signals with various types of noise added (ECG plus HF, LF, PL, and IM noise, respectively) were analyzed. Then, the effects of noise, signal length, and signal-to-noise ratio (SNR) on the LZ complexity of ECG signals were analyzed. The simulation results show that LZ complexity for HF noise was obviously different from those for PL and LF noise. The LZ value can be used to determine the presence of HF noise. ECG plus HF noise had the highest LZ values. Other types of noise had low LZ values. Signal lengths of over 40 s had only a small effect on LZ values. The LZ values for ECG plus all types of noise increased monotonically with decreasing SNR except for LF and PL noise. For the test of real ECG signals plus three types of noise, namely muscle artefacts (MAs), baseline wander (BW), and electrode motion (EM) artefacts, LZ complexity varied obviously with increasing MA but not for BW and EM noise. This study demonstrates that LZ complexity is sensitive to noise level (especially for HF noise) and can thus be a valuable reference index for the assessment of ECG signal quality.

## Introduction

Electrocardiography (ECG) recordings are often contaminated by various types of noise, including movement artefacts, power-line (50 or 60 Hz) noise, and muscular electrical activity. The presence of this noise can render the signals unsuitable for clinical use, wasting the resources utilized for their acquisition [1]. Assessing the quality of ECG signals would be extremely helpful with this regard.

The increasing use of mobile devices for acquiring ECG signals has recently driven research interest towards the problem of assessing the signal quality of ECG recordings [2–5]. Existing methods are mostly based on the characterization of time or frequency features of the signals. Time-based methods aim to identify particular characteristics, such as RR time interval outliers [6], flat lines, baseline wander (BW), and steep slopes [7], which usually can compromise recordings. Frequency-based methods use, for example, the ratio between the low- and high-frequency power of the signal [8]. A method that combines time and frequency information has also been proposed [9, 10]. Non-linear methods, such as entropy measures, have also been explored for this application but need further investigation [11, 12].

Lempel–Ziv (LZ) complexity [13–15] is a measure of the complexity of a signal. It has been applied to a variety of biomedical signals, including ECG from patients with ventricular tachycardia or atrial fibrillation [16, 17], heart sound signals from patients with cardiovascular disease [18], electroencephalograms (EEG) from patients with Alzheimer’s disease [19, 20], EEG sleep signals [21], and brain function [22]. Aboy et al. [23] analysed the LZ complexity of periodic signals, Gaussian white noise, and colored noise. However, how typical types of noise affect the LZ complexity of ECG recordings, and the relationship between the LZ complexity and noise level, have not yet been systematically studied.

The aim of this study was to characterize the LZ complexity of various ECG signals (noise-free and noisy) and to assess the effects of noise type on the LZ complexity of ECG signals.

## Materials and Methods

### Database

#### Artificial ECG Signals

Clean artificial ECG signals were generated using the open source ECGSYN software, as described by McSharry et al. [24]. The sample rate was set at 360 Hz. The heart rate was set to be within the range of 50–100 beats per minute (bpm). Four types of noise were separately added to the clean ECG signals: 50–180 Hz high-frequency (HF) noise; 50 Hz power-line (PL) noise; 0–0.5 Hz low-frequency (LF) noise; and impulse (IM) noise. HF noise was used to simulate muscular electrical activity and other HF noise. In order to avoid the overlap of the frequency range of HF noise and the main frequency range of ECG, 50–180 Hz was chosen (the upper end of the range was determined at a sample rate of 360 Hz). PL noise was used to simulate noise from the mains. LF noise was used to simulate BW because its frequency range overlaps with 0–0.5 Hz [25, 26]; it can approximately be regarded as an electrode motion artefact with a significant amount of BW [27]. IM noise was used to simulate the spikes with high amplitudes contained in ECG signals. To generate IM noise, the zero sequence was replaced by various percentages of random spikes. To generate HF noise, Gaussian noise was firstly generated and then filtered by a band-pass filter (50–180 Hz). To generate LF noise, Gaussian white noise was firstly generated and then filtered by a low-pass filter (0–0.5 Hz).

#### Real ECG Signals

Real ECG signals were selected from the Massachusetts Institute of Technology Beth Israel Hospital (now the Beth Israel Deaconess Medical Centre) (MIT/BIH) arrhythmia database [28, 29]. This database contains 48 ECG recordings, each with a duration of 30 min and a sample rate of 360 Hz. Baseline correction for removing the main LW noise was performed because the BW noise contained in the raw signals could lead to inaccurate results. The processed ECG signals were used for further study.

Real noise signals taken from the Noise Stress Test Database (NSTDB) [30] were used. NSTDB provides three types of noise that can be typically found in ambulatory ECG recordings: muscle artefacts (MA), electrode motion (EM), and BW. Because NSTDB does not include 50-Hz PL noise and IM noise, this study added these types of noise to the real ECG signals for testing.

Both the MIT-BIH database and NSTDB are publicly available through the Physionet website [29].

### LZ Complexity

Before LZ complexity can be computed, the original signal must be coarse-grained, and then transformed into a symbols sequence for simplifying the computation. In previous works, the binary (two-state) sequence was demonstrated to adequately represent the LZ complexity of the original signal [16, 23, 31]. For generating the two-state sequence, the signal data were converted into a 0–1 sequence *R* by comparison with the threshold *T*_*h*_. The binary symbolic sequence *R* = {*r*(1), *r*(2),…, *r*(*n*)} was produced as follows:

1 $$r(i) = \left\{ {\begin{array}{*{20}c} {0,\,\,\,if\,\,x(i) < T_{h} } \\ {1,\,\,\,if\,x(i) \ge T_{h} } \\ \end{array} } \right.,\quad i = \,1,2 \cdots n$$

where *n* is the length of *x*(*n*). Usually, the mean value of the sequence is used as the threshold *T*_*h*_ [16, 32]. This was thus done for the coarse-graining process in this study.

Following the initial coarse-graining process, the LZ complexity *c*(*n*) for the symbol sequence *R* was computed. The whole binary sequence *R* is scanned from left to right, and the counter *c*(*n*) is increased by one unit when a new subsequence (a new pattern) of consecutive characters is encountered in the scanning process. The counter *c*(*n*) conforms to the following rules [16, 23, 29]:

1. Let *S* and *Q* denote two strings, respectively, *SQ* be the concatenation of *S* and *Q*, string *SQπ* be derived from *SQ* after its last character is deleted (*π* means the operation to delete the last character in the string). Let *v*(*SQπ*) denote the vocabulary of all different substrings of *SQπ*. Initially, *c*(*n*) = 1, *S* = *s*_1_, and *Q* = *s*_2_, and so *SQπ* = *s*_1_.
2. In summary, *S* = *s*_1_ *s*_2_, …, *s*_*r*_, *Q* = *s*_*r*+1_, and so *SQπ* = *s*_1_ *s*_2_, …, *s*; if *Q* belongs to v(*SQπ*), then *s*_*r*+1_, that is, *Q* is a substring of *SQπ*, and so *S* does not change. Update *Q* to be *s*_*r*+1_ *s*_*r*+2_, and then judge whether *Q* belongs to *v*(*SQπ*). Repeat this process until *Q* does not belong to *v*(*SQπ*).
3. Now, *Q* = *s*_*r*+1_ *s*_*r*+2_, …, *s*_*r*+*i*_, which is not a substring of *SQπ* = *s*_1_ *s*_2_, …, *s*_*r*_ *s*_*r*+1_,…, *s*_*r*+*i*-1_, so increase *c*(*n*) by one.
4. Thereafter, *S* is updated to be *S* = *s*_1_ *s*_2_, …, *s*_*r*+*i*_, and *Q* = *s*_*r*+*i*+1_.

Then, the procedure is repeated until *Q* is the last character. At this time, the counter *c*(*n*) is the number of different substrings contained in *R*, and it reflects the number of different patterns in a sequence. *c*(*n*) might vary with sequence length [17, 23]. Thus, in order to obtain a complexity measure independent of the sequence length, *c*(*n*) should be normalized [17, 23].

It has been proved that the upper bound of *c*(*n*) is:

2 $$c(n) < \frac{n}{{(1 - \varepsilon_{n} )\log_{\alpha } (n)}}$$

where *n* is the length of the sequence and *α* is the number of different symbols in the symbol set. In this study, *α* was 2 because the coarse-grained sequence was a 0–1 sequence. *ε*_*n*_ is a small quantity and *ε*_*n*_ → 0 (n → ∞). In fact:

3 $$\mathop {\lim }\limits_{n \to \infty } c(n) = b(n) = \frac{n}{{\log_{\alpha } (n)}}$$

*c*(*n*) can be normalized as:

4 $$C(n) = \frac{c(n)}{b(n)}$$

where *C*(*n*) is the normalized LZ complexity, and denotes the arising rate of new patterns within the sequence. A detailed LZ complexity analysis can be found elsewhere [23]. In this study, the normalized complexity *C*(*n*), rather than *c*(*n*), is regarded as the result of LZ complexity.

### LZ Complexity Analysis for ECG Signals

#### LZ Analysis for Typical Signals

The LZ complexity was calculated separately for some typical signals, specifically various types of artificial signal (HF, LF, PL, and IM noise and clean ECG) and noisy ECG signals. In this test, IM noise was generated by replacing 10 % of the 40-s zero sequence with random spikes. For each type of signal, 50 repeats were used to reduce the effect of random factors. Each repeat lasted for 40 s. For the synthetic noisy ECG signal, the signal-to-noise ratio (SNR) is defined as:

5 $$SNR = 10 \times \log_{10} \left( {{\raise0.7ex\hbox{${P_{signal} }$} \!\mathord{\left/ {\vphantom {{P_{signal} } {P_{noise} }}}\right.\kern-0pt} \!\lower0.7ex\hbox{${P_{noise} }$}}} \right)$$

where *P*_*signal*_ and *P*_*noise*_ denote the power of the clean ECG and that of the noise, respectively.

In this test, the SNR of the synthetic ECG was set as 10 dB. By analysing the LZ values of these signals and their statistics (i.e., the mean and standard deviation), we tried to show typical LZ complexity values for the special types of signals.

#### Effect of Signal Length on LZ Complexity

The effect of signal length on LZ complexity was tested using the five types of signals: clean ECG signals and synthetic ECG signals plus HF, PL, LF, and IM noise, respectively. For each type of signal, the SNR of 10 dB was used and the signal length was varied from 5 to 120 s, in steps of 5 s. For each signal length, 50 repeats were performed and the mean values and standard deviations were determined for comparison. This effect analysis aimed to determine a suitable signal length to obtain stable LZvalues.

#### Effect of SNR on LZ Complexity

In this test, SNR was varied from −10 to 20 dB for each type of signal, in steps of 5 dB. For each SNR level, 50 repeats were produced and the mean values and standard deviations were calculated. To observe the mixed effect of the various types of noise, all noise types were added to the clean ECG signals. The mixed noise included HF, PL, LF, and IM noise, with the same proportion (25 %) for each noise type. For the SNR effect analysis, the optimal signal length from the effect analysis of signal length was used.

**Fig. 1:**
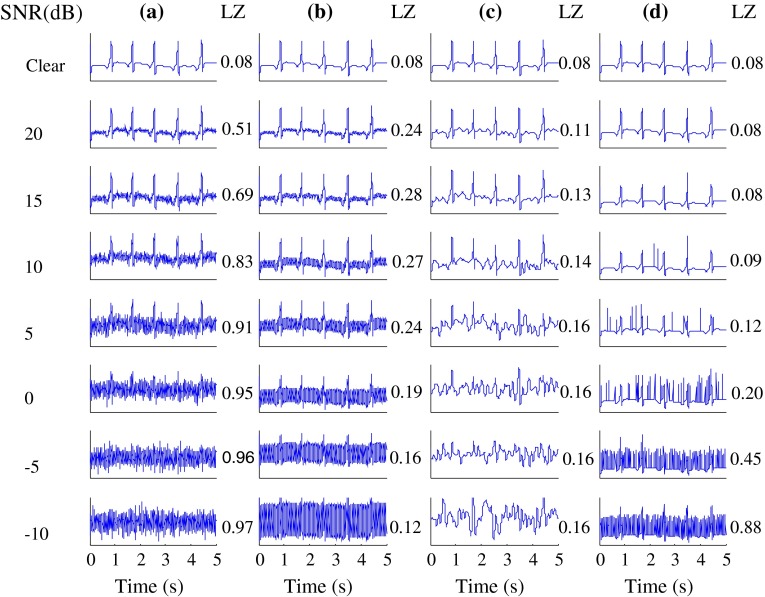
Clean artificial ECG signals and these signals plus various types of noise at various SNR levels (−10 to 20 dB, in steps of 5 dB). Artificial ECG signals plus **a** HF, **b** PL, **c** LF and **d** IM noise. Corresponding LZ values are also shown

**Fig. 2:**
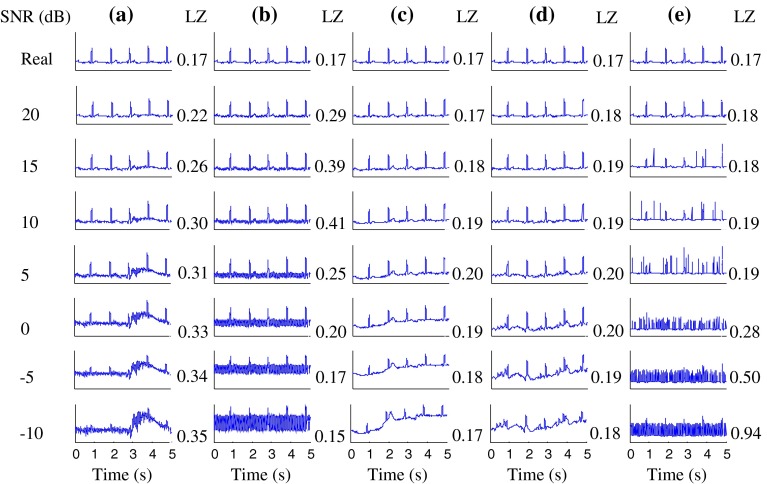
Real ECG signals and these signals plus various types of noise at various SNR levels (−10 to 20 dB, in steps of 5 dB). Real ECG signals plus **a** MA, **b** PL, **c** BW, **d** EM and **e** IM noise. Corresponding LZ values are also shown

Figure 1 gives examples of the artificial ECG signals, namely the clean ECG signals and the synthetic noisy ECG signals at various SNR levels, as well as their LZ values. Figure 2 gives similar examples from the real ECG signals. It is can be seen that the identifiability of ECG waveforms decreases with decreasing SNR.

#### Receiver Operating Characteristic Curve for Verification of LZ Complexity

For verification, we used the receiver operating characteristic (ROC) curve to evaluate the performance of LZ complexity for signal quality assessment. According to pre-observations, the clean artificial ECG signals plus HF, LF, or PL noise were identified as too noisy (unacceptable) for clinical application when SNR was less than 5 dB. Similarly, the threshold for the clean artificial ECG signals plus IM noise was set as 0 dB. We set the threshold of 4.6 dB for classifying the clear artificial ECG plus mixed noise as “unacceptable” (SNR <= 4.6 dB) or “acceptable” (SNR > 4.6 dB) in this study. The reason for choosing these values is that the main waveform features (i.e., P, Q, and T waves) of the corrupted ECGs could be identified when SNR decreases below the specified thresholds. For the mixed-noise-corrupted ECG, we observed that an SNR value of between 4 and 5 dB can be used as the threshold. A threshold of 4.6 dB was thus chosen based on the ROC analysis.

## Results

### LZ Values for Typical ECG Signals

**Fig. 3:**
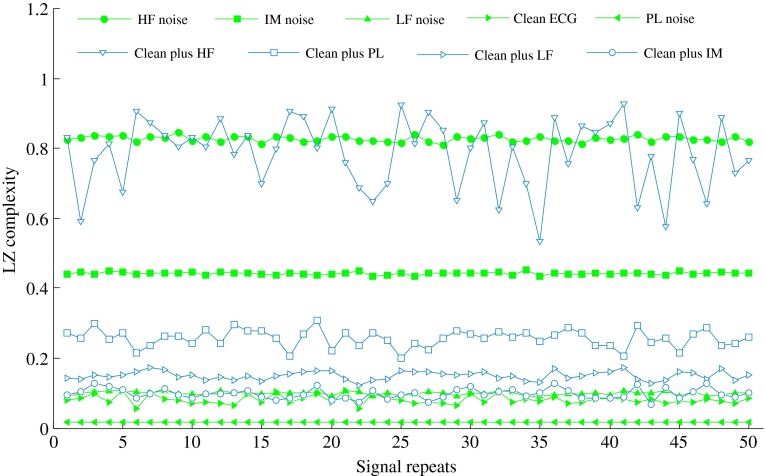
LZ complexity results from typical signals when performing 50 repeat calculations with signal length of 40 s and SNR of 10 dB

Figure 3 shows the LZ results of the typical signals (i.e., HF, LF, PL, and IM noise, the clean ECG, and the clean ECG plus HF, PL, LF, and IM noise, respectively) when performing 50 repeat calculations for each type of signal. HF noise showed the highest values of LZ complexity. LF noise had an LZ value of close to 0.1, and the clean ECG had a slightly lower LZ than that of LF noise. PL noise had the lowest LZ value.

We also calculated the mean values and standard deviations of the 50 repeats for each type of signal with SNR set to 10 dB. These values are shown in Table 1.

**Table 1:** Mean values and standard deviations of LZ complexity from 50 repeats for each type of signal

Signal type	Mean	Standard deviation
HF noise	0.8278	0.0082
LF noise	0.0994	0.0051
PL noise	0.0163	0.0000
IM noise	0.4425	0.0039
Clean ECG	0.0807	0.0124
Clean plus HF noise	0.7873	0.1010
Clean plus PL noise	0.2567	0.0249
Clean plus LF noise	0.1502	0.0122
Clean plus IM noise	0.0983	0.0245

### Effect of Signal Length

**Fig. 4:**
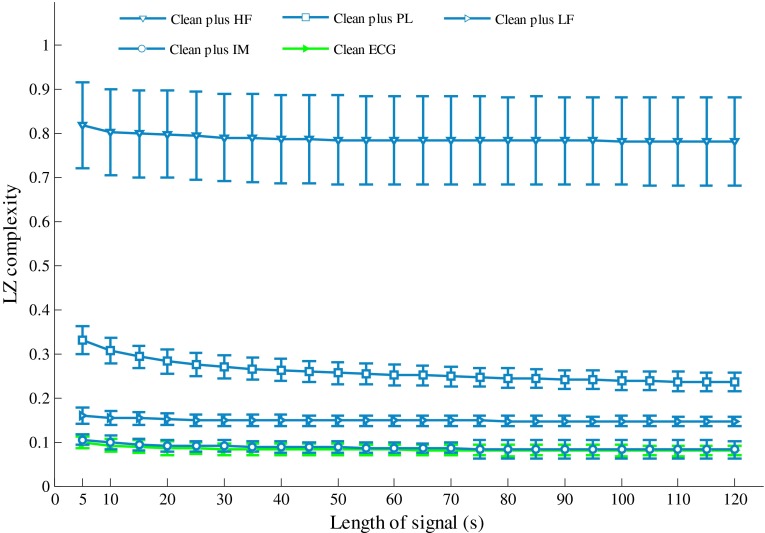
Effect of signal length on LZ complexity for artificial noisy ECG signals

Figure 4 shows the effects of signal length on LZ complexity for the artificial ECG signals. The LZ complexity decreased with increasing signal length when the signal length was shorter than 20 s. When the signal length was longer than 40 s, the LZ complexity remained stable for all of signal types. Therefore, a signal length of 40 s was used for the following analysis.

### Effect of SNR

**Fig. 5:**
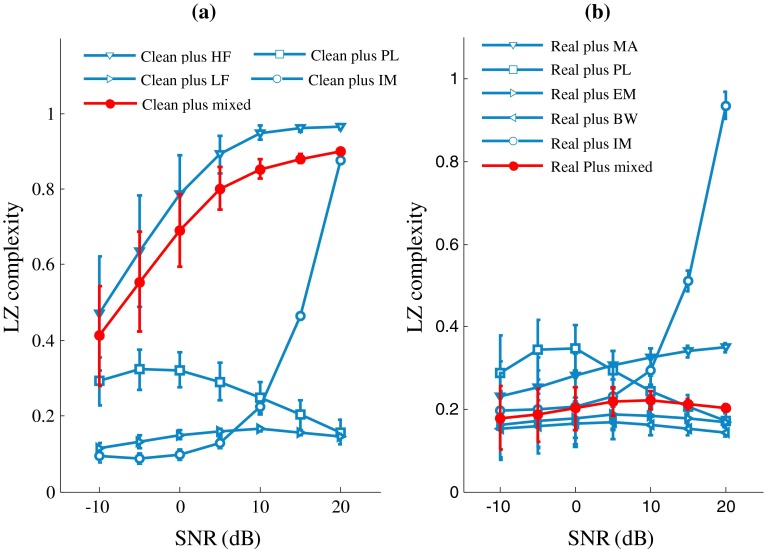
Effect of SNR on LZ complexity for **a** artificial and **b** real noisy ECG signals

Figure 5 shows the effects of SNR on LZ complexity for both the artificial (Fig. 5a) and real (Fig. 5b) ECG signals. As shown in Fig. 5a, the LZ values of the clean ECG plus HF or IM noise increase quickly and monotonically with decreasing SNR. The LZ values of the clean ECG plus PL noise increase until SNR reaches 10 dB and then decrease. The LZ values from the clean ECG plus LF noise decrease when the SNR is below 0 dB. Figure 5b shows the change trend of the LZ values for the real ECG signals. The LZ values from the real ECG plus MA or IM noise increase monotonously with decreasing SNR, and the other types of signals first increase and then decrease.

**Fig. 6:**
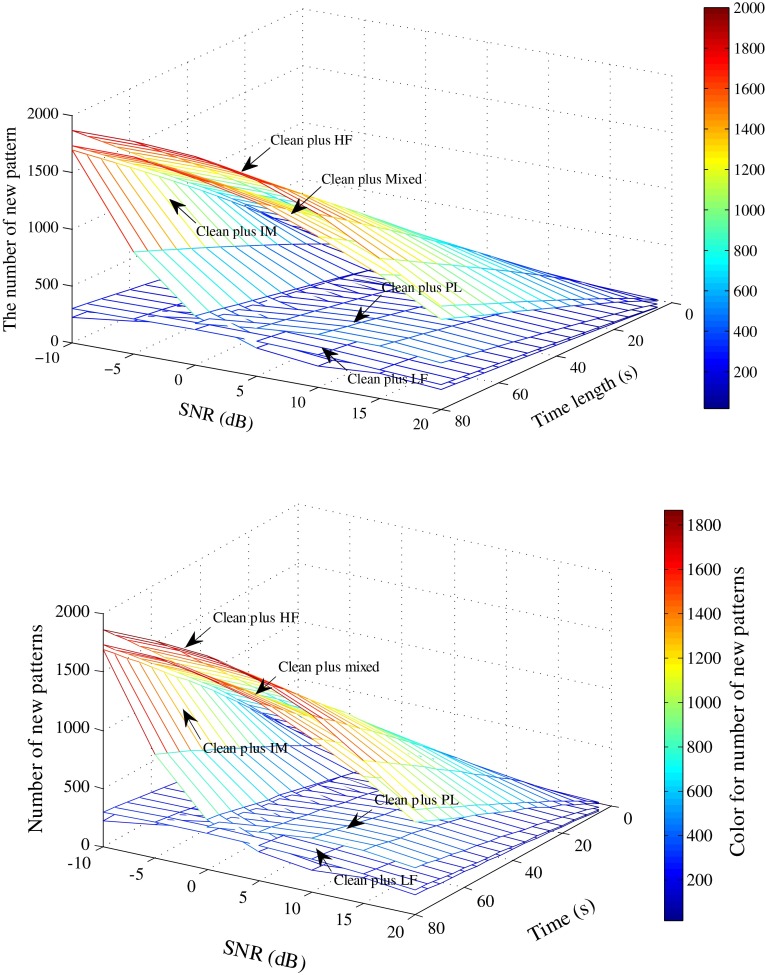
Number of new patterns of artificial ECG plus HF, PL, LF, IM, and mixed-type noise, respectively, at various signal length and SNR settings

In order to further explain the effect of SNR on LZ complexity, we analyzed the relationship between SNR and the number of new patterns for the clean artificial ECG signals plus five types of noise (i.e., HF, PL, LF, IM, and mixed noise). Figure 6 shows the results. It is clear that the number of new patterns from the ECG plus HF, IM, or mixed noise significantly increase with increasing signal length and decreasing SNR, whereas the other LZ values do not show obvious changes. The change trends of the number of new patterns of five synthetic signals with decreasing SNR are consistent with the LZ values of these signals. It is also worth to note that for the clean ECG plus LF or PL noise, the number of new patterns with an SNR of −10 dB is lower than that with an SNR of 0 dB. This is because many signal details are lost during the coarse-graining process and the number of new patterns begins to drop off when SNR decreases to a certain level.

The one-way analysis of variance (ANOVA) was also emplyed for analysing the effects of the mixed noise types at various SNR values (20, 15, 10, 5, 0, −5, and −10 dB) on LZ complexity. The ANOVA results (F = 243.709, P = 0.000) indicate that the LZ complexities of the synthetic ECG at various SNR values have significant differences.

### Validation Using ROC Curve

The Youden index (YI) was employed for choosing the optimal threshold. It is defined as follows:

6 $${\text{YI}}\, = \,{\text{Sensitivity}} + {\text{Specificity}} - \,1$$

**Fig. 7:**
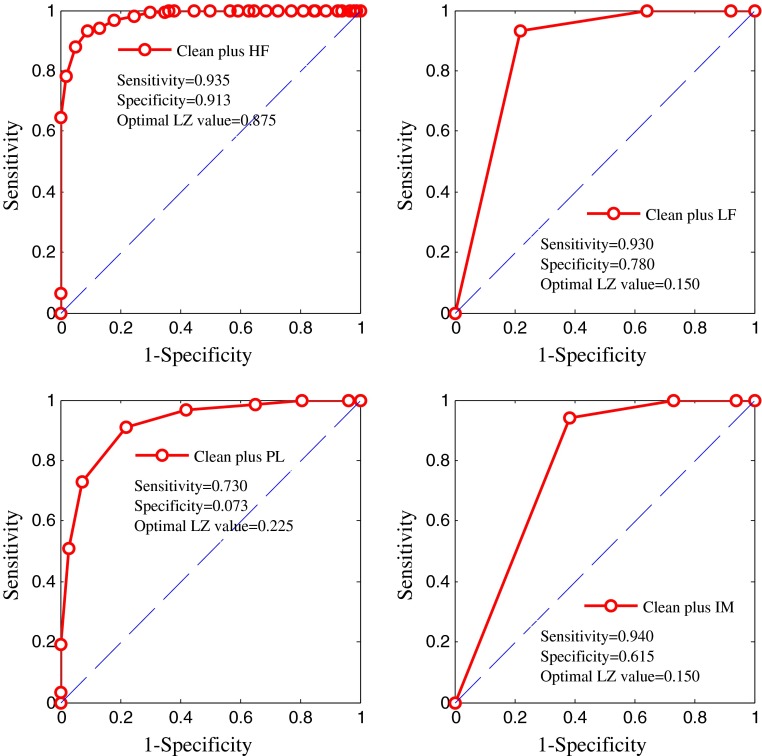
ROC curve for artificial ECG plus **a** HF, **b** PL, **c** LF and **d** IM noise

The ROC curve of LZ complexity was used to evaluate classification performance. Figure 7 shows the ROC curve of the LZ values of four synthetic ECGs, i.e., the clean ECG plus HF, LF, PL, and IM noise, respectively. The optimal LZ threshold for the clean ECG plus HF noise is 0.875, those for the clean ECG plus LF and IM noise are 0.150, and that for the clean ECG plus PL noise is 0.225.

**Fig. 8:**
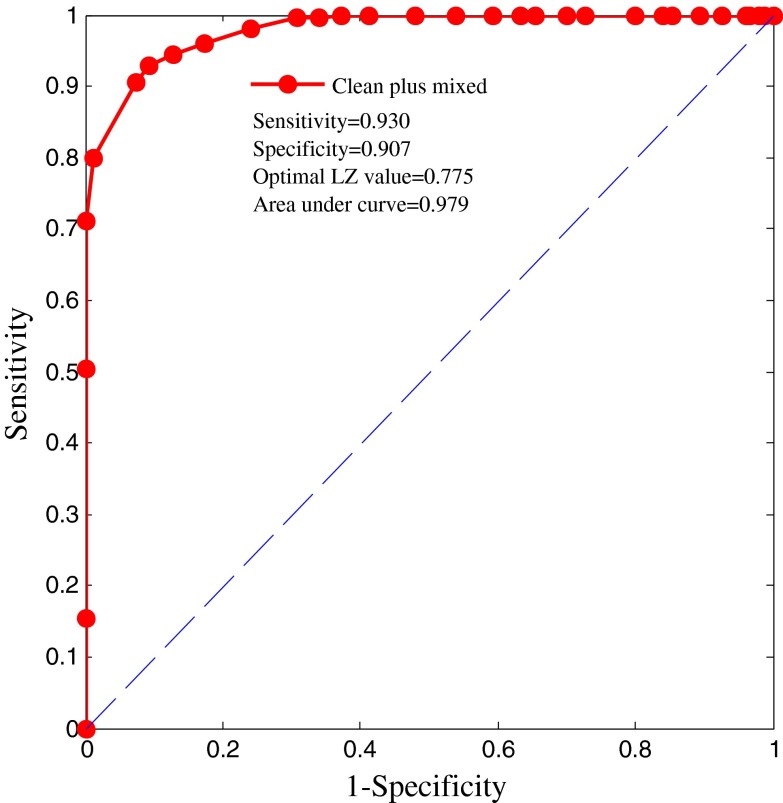
ROC curve for artificial ECG plus mixed-type noise

Figure 8 shows the ROC curve of the mixed-noise-corrupted ECG. The area under the curve is equal to 0.979, which means that the classification performance of the LZ complexity is good. The optimal LZ complexity (cut-off value) is equal to 0.775 for the artificial synthetic ECG plus mixed noise. Table 2 shows the sensitivity, specificity, and YI values for given LZ complexity thresholds for the mixed-noise-corrupted ECG.

**Table 2:** Sensitivity, specificity, and YI values for given LZ complexity thresholds for mixed-noise-corrupted ECG

LZ threshold	0.000	0.025	0.050	0.075	0.100	0.125	0.150	0.175	0.200	0.225	0.250	0.275	0.300	0.325
Sensitivity	1.000	1.000	1.000	1.000	0.000	1.000	1.000	1.000	0.000	1.000	1.000	1.000	1.000	1.000
Specificity	0.000	0.000	0.000	0.000	0.000	0.000	0.000	0.013	0.020	0.033	0.033	0.040	0.073	0.106
YI	0.000	0.000	0.000	0.000	−1.000	0.000	0.000	0.013	−0.980	0.033	0.033	0.040	0.073	0.106

LZ threshold	0.350	0.375	0.400	0.425	0.450	0.475	0.500	0.525	0.550	0.575	0.600	0.625	0.650	0.675
Sensitivity	1.000	1.000	1.000	1.000	1.000	1.000	1.000	1.000	1.000	1.000	1.000	1.000	0.995	0.995
Specificity	0.147	0.160	0.200	0.273	0.300	0.347	0.367	0.407	0.460	0.520	0.587	0.627	0.660	0.693
YI	0.147	0.160	0.200	0.273	0.300	0.347	0.367	0.407	0.460	0.520	0.587	0.627	0.655	0.688

LZ threshold	0.700	0.725	0.750	**0.775**	0.800	0.825	0.850	0.875	0.900	0.925	0.950	0.975	1.000
Sensitivity	0.980	0.960	0.945	**0.930**	0.905	0.800	0.710	0.505	0.155	0.000	0.000	0.000	0.000
Specificity	0.760	0.827	0.873	**0.907**	0.927	0.987	1.000	1.000	1.000	1.000	1.000	1.000	1.000
YI	0.740	0.787	0.818	**0.837**	0.832	0.787	0.710	0.505	0.155	0.000	0.000	0.000	0.000

Bold values indicate the highest YI index achieved

## Discussion

In this study, we systematically characterized the values of LZ complexity when different types of noise affected the ECG recordings. Figure 3 shows that the different signal types have different LZ values. In general, the LZ complexity is stable, except for the clean ECG signal plus HF noise, which shows fluctuation. The LZ values for the typical signals indicate that the LZ complexity is not only closely associated with the periodicity or randomness of the signals, but is also significantly different between various types of noise. The LZ complexity is low when the signal has an obvious periodicity. The ROC curve analysis shows that the classification performance of the LZ complexity is good, especially for the HF-noise-corrupted ECG.

This study showed that the LZ complexity can indicate the noise level contained in ECG signals. LZ complexity can thus be applied as a metric for assessing the quality of ECG signals corrupted by various types of noise. For ECG signals corrupted by LF noise, we recommend that the baseline should be removed before determining LZ complexity. This study also tested the performance of LZ complexity for real ECG signals. The change trends were consistent with the results for the artificial ECG.

## Conclusion

Application of Lempel–Ziv (LZ) complexity for ECG quality assessment was investigated in this study and it concluded that LZ complexity is sensitive to noise level (especially for HF noise) and can thus be a valuable reference index for the assessment of ECG signal quality.
